# Partial and Radical Nephrectomy Provides Equivalent Oncologic Outcomes in pT3a Renal Cell Carcinoma: A Population-Based Study

**DOI:** 10.3389/fonc.2021.819098

**Published:** 2022-01-26

**Authors:** Jihua Tian, Xing Zeng, Jie Wan, Jiahua Gan, Chunjin Ke, Wei Guan, Zhiquan Hu, Chunguang Yang

**Affiliations:** ^1^ Department of Urology, Tongji Hospital of Tongji Medical College, Huazhong University of Science and Technology (HUST), Wuhan, China; ^2^ Department of Pathology, Tongji Hospital of Tongji Medical College, Huazhong University of Science and Technology, Wuhan, China

**Keywords:** partial nephrectomy, radical nephrectomy, renal cell carcinoma, outcomes, T3a

## Abstract

**Purpose:**

To compare the cause-specific survival (CSS) and overall survival (OS) of patients with localized T3a renal cell carcinoma (RCC) after partial nephrectomy (PN) or radical nephrectomy (RN).

**Methods:**

We obtained the demographic and clinicopathological data of 7,127 patients with localized T3a RCC and who underwent PN or RN from the Surveillance, Epidemiology, and End Results (SEER) database. These patients were divided into fat invasion cohort and venous invasion cohort for subsequent analysis. Kaplan–Meier analysis (KMA) and univariate and multivariate Cox proportional hazards regression analyses were used to evaluate the effects of PN or RN on OS and CSS. Meanwhile, 65 cases with clinical T1 (cT1) RCC upstaged to pathological T3a (pT3a) who were treated in Tongji Hospital (TJH) from 2011 to 2020 and underwent PN or RN were identified.

**Results:**

In the study cohort, 2,085 (29.3%) patients died during the 1–172 months’ follow-up, of whom 1,155 (16.2%) died of RCC. In the two cohorts of fat invasion and venous invasion, KMA indicated that the PN group had favorable survival (*p* < 0.001). However, after propensity score matching (PSM), univariate and multivariate Cox regression analyses showed that the PN and RN groups had comparable CSS in the fat invasion cohort (*p* = 0.075) and the venous invasion cohort (*p* = 0.190). During 1–104 months of follow-up, 9 cases in the Tongji cohort had disease recurrence. There was no significant difference in recurrence-free survival between the RN group and the PN group (*p* = 0.170).

**Conclusions:**

Our analysis showed that after balancing these factors, patients with localized pT3a RCC receiving PN or RN can achieve comparable oncologic outcomes. PN is safe for selected T3a patients.

## Introduction

Renal cell carcinoma (RCC) accounts for 2%–3% of all adult malignancies (1). In recent years, with the wide applications of imaging examinations, the proportion of early-staged RCC has gradually increased (2), and partial nephrectomy (PN) has therefore played a more important role in the treatment of RCC (3, 4).

PN is currently the standard treatment for T1 RCC, which provides similar oncologic control to radical nephrectomy (RN) while reducing the loss of renal function (5, 6). The current guidelines for PN recommendations are limited to T1 and selected T2 RCC (combined with solitary kidney or chronic kidney disease if technically feasible) (5, 7, 8). However, in clinical practice, patients with clinical T1 (cT1) upstaged to pathological T3a (pT3a) RCC underwent PN did not show unfavorable cause-specific survival (CSS) and recurrence-free survival (RFS) than did those who received RN, which led us to think about the safety of PN for selected T3a patients (9, 10).

In the past 10 years, the application of PN in T3a RCC patients has been explored, more and more evidences show that PN is safe and feasible for some T3a cases (10–13), and there are also dissenting voices suggesting that PN is associated with poor oncologic outcome (14). Most of these studies are small-volume and retrospective. To this end, we selected the Surveillance, Epidemiology, and End Results (SEER) database to compare the performance of PN and RN in T3a RCC patients and used propensity score matching (PSM) to control bias; finally, we attached data of cT1 RCC patients upstaged to pT3a from our institution to add new evidence to this controversy.

## Patients and Methods

**Figure 1:**
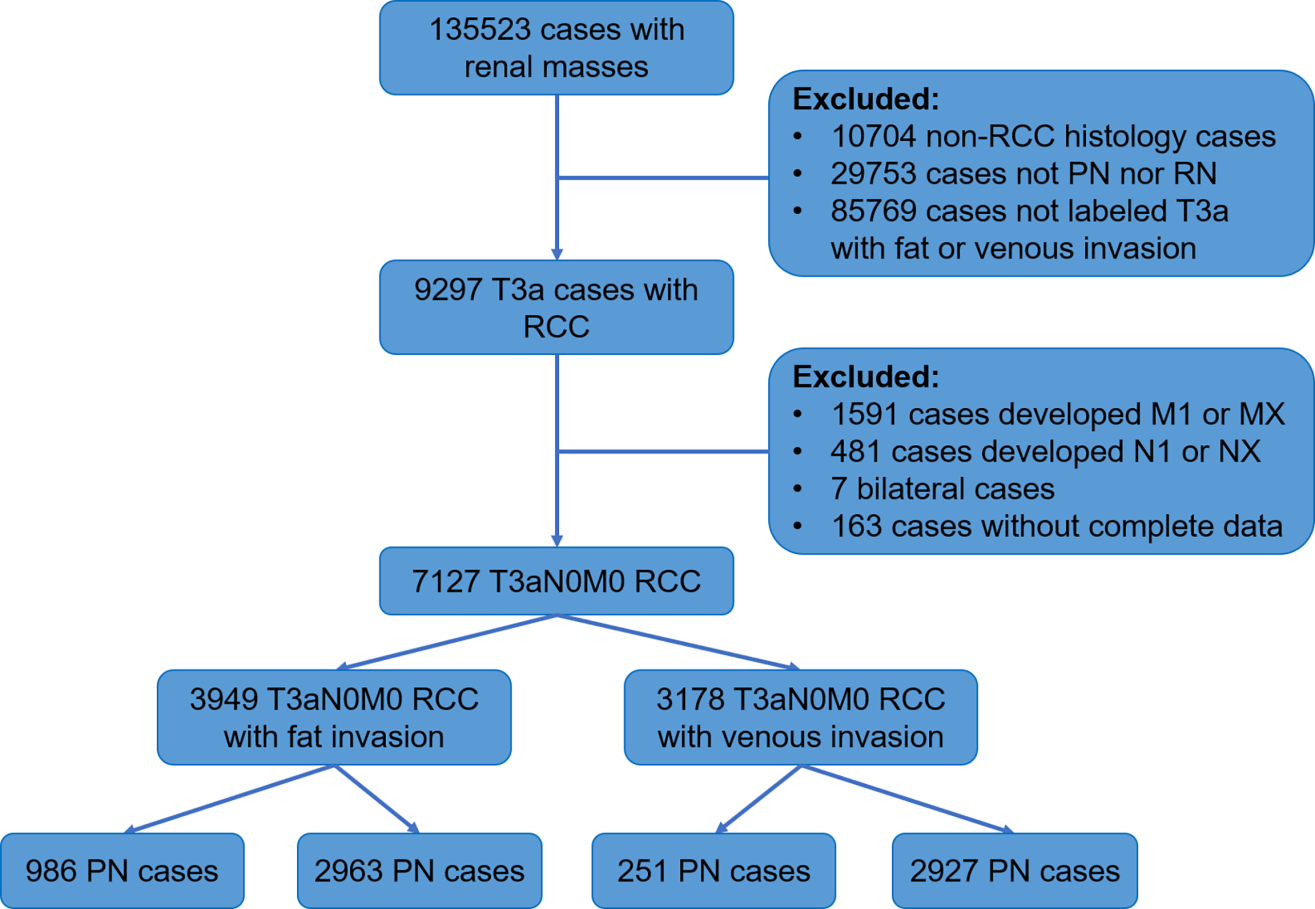
Flowchart displaying patient selection procedure.

SEER is a population-based cancer database found by the National Cancer Institute that collects data on morbidity, treatment, and mortality (15). We obtained data of RCC patients diagnosed from 2004 to 2015 from 18 registries of the SEER database. The process of screening patients was shown below (**Figure 1**).

The inclusion criteria of the study were as follows: malignant pathological diagnosis; age 15 years and above; histologic type is renal cancer (including clear cell RCC (CCRCC), RCC not otherwise specified (NOS), papillary RCC (PRCC), acquired cystic disease-associated RCC/tubulocystic RCC, chromophobe RCC (CHRCC), clear cell PRCC, collecting duct carcinoma, hereditary leiomyomatosis and RCC-associated RCC, MiT family translocation RCC, mucinous tubular and spindle cell carcinoma, and renal medullary carcinoma); staged T3a; no lymph nodes and distant metastasis; no bilateral or other tumors; the surgical method was PN or RN; follow-up longer than 1 month; and knowable survival status.

The histologic types were divided into CCRCC, PRCC, CHRCC, and Others. According to the tissues of tumor invasion, the study cohort was divided into the fat invasion and venous invasion cohorts. The demographic and clinicopathological data of the RN and PN groups in the two cohorts were analyzed separately. The continuous variables were analyzed by Student’s t-test, and the categorical variables were analyzed by Fisher’s exact test. Then, a 1:1 PSM was performed between the surgical methods (PN vs. RN); all demographic and clinicopathological features were used as calipers (half of the PN cases in the fat invasion cohort were randomly rejected because of lack of enough RN cases to match).

The Kaplan–Meier analysis (KMA) was used to evaluate the survival difference between the RN and PN groups in the venous invasion cohort and the fat invasion cohort, respectively. Then univariate and multivariate Cox regression analyses were used to analyze the effect of each factor on survival. The variables of multivariate Cox regression were derived from the significant prognostic factors of univariate Cox regression.

Finally, after institutional review board approval, a retrospective review of all patients with upstaged pT3a RCC managed with PN or RN (2010.1.1–2020.12.1) in the Department of Urology of Tongji Hospital of Tongji Medical College, Huazhong University of Science and Technology (HUST) was performed (n = 65). KMA and univariate and multivariate Cox regression analyses were also performed in this cohort. All the above analyses were performed by R version 4.0.3 (Institute for Statistics and Mathematics, Vienna, Austria; www.r-project.org), and *p* < 0.05 was considered statistically significant.

## Results

After the screening procedure, 7,127 T3aN0M0 RCC cases (**Table 1**) were obtained. The median age was 62 years (22–87 years). There were 1,237 cases (17.4%) who received PN. The maximum diameter of tumors in the PN group was significantly smaller than that of the RN group (40 [29.00, 53.00] mm vs. 74 [55.00, 95.00] mm, *p* < 0.001). There were also significant differences in histologic types and nuclear grade structure between the two groups (both *p* < 0.001). The proportion of CCRCC and high-grade RCC in the RN group was higher. The median follow-up time was 56 months (1–172 months); 2,085 cases (29.3%) were confirmed dead at the last follow-up, of whom 1,205 cases (60.4%) died of RCC; the 1-, 3-, and 5-year survival rates were 95.1%, 83.4%, and 73.8%, respectively.

**Table 1:** Characteristics of PN and RN groups in SEER cohorts.

Characteristics	PN (n = 1,237)	RN (n = 5,890)	*p*
Year of diagnosis (median [IQR])	2,013.00 [2,011.00, 2,014.00]	2,013.00 [2,011.00, 2,014.00]	<0.001
Age (median [IQR])	62.00 [57.00, 72.00]	62.00 [57.00, 72.00]	0.013
Size (median [IQR])	40.00 [29.00, 53.00]	74.00 [55.00, 95.00]	<0.001
Grade (%)			<0.001
I	68 (5.5)	166 (2.8)	
II	508 (41.1)	1,946 (33.0)	
III	414 (33.5)	2,303 (39.1)	
IV	73 (5.9)	763 (13.0)	
Unknown	174 (14.1)	712 (12.1)	
Race (%)			0.012
Black	111 (9.0)	397 (6.7)	
Other	88 (7.1)	377 (6.4)	
White	1,038 (83.9)	5,116 (86.9)	
Sex (%)			0.162
Female	352 (28.5)	1,796 (30.5)	
Male	885 (71.5)	4,094 (69.5)	
Histology (%)			<0.001
CCRCC	766 (61.9)	4,544 (77.1)	
CHRCC	119 (9.6)	312 (5.3)	
PRCC	238 (19.2)	323 (5.5)	
Others	114 (9.2)	721 (12.2)	
Laterality (%)			0.080
Left	598 (48.3)	3,010 (51.1)	
Right	639 (51.7)	2,880 (48.9)	
Chemotherapy (%)			<0.001
No/unknown	1,215 (98.2)	5,609 (95.2)	
Yes	22 (1.8)	281 (4.8)	
Extension (%)			<0.001
Fat	986 (79.7)	2,963 (50.3)	
VTT	251 (20.3)	2,927 (49.7)	

PN, partial nephrectomy; RN, radical nephrectomy; SEER, Surveillance, Epidemiology, and End Results; IQR, interquartile range; CCRCC, clear cell renal cell carcinoma; CHRCC, chromophobe renal cell carcinoma; PRCC, papillary renal cell carcinoma; VTT, venous tumor thrombus.

**Figure 2:**
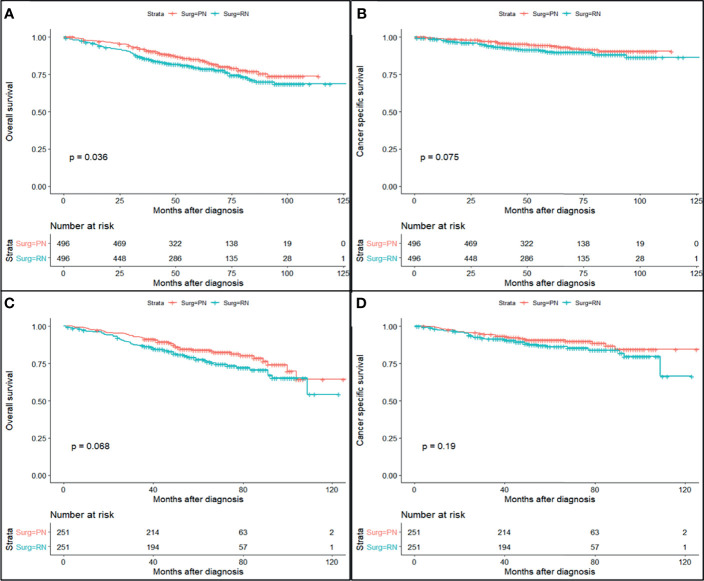
Survival curve of partial nephrectomy (PN) and radical nephrectomy (RN) group in fat invasion cohort and venous invasion cohort after propensity score matching (PSM). **(A)** Overall survival (OS) curve of fat invasion cohort. **(B)** Cause-specific survival (CSS) curve of fat invasion cohort. **(C)** OS curve of venous invasion cohort. **(D)** CSS curve of venous invasion cohort.

**Figure 3:**
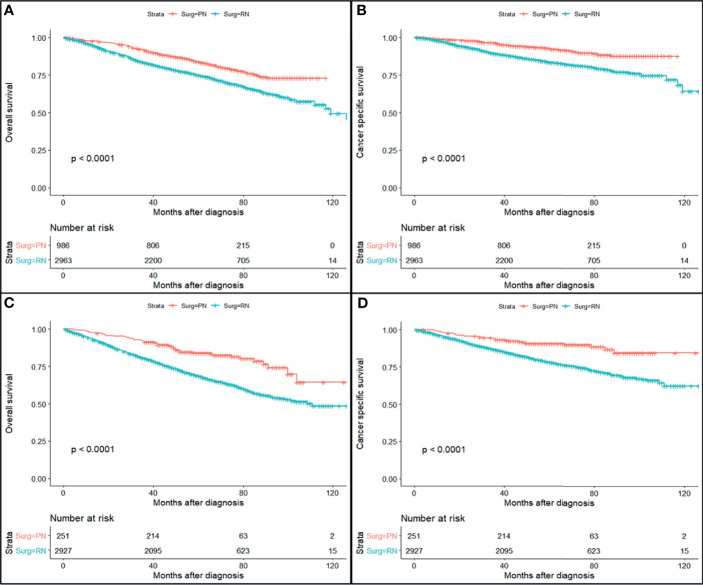
Survival curve of partial nephrectomy (PN) and radical nephrectomy (RN) group in fat invasion cohort and venous invasion cohort before propensity score matching (PSM). **(A)** Overall survival (OS) curve of fat invasion cohort. **(B)** Cause-specific survival (CSS) curve of fat invasion cohort. **(C)** OS curve of venous invasion cohort. **(D)** CSS curve of venous invasion cohort.

The study cohort was divided into 3,949 cases of fat invasion (including 986 cases of PN and 2,963 cases of RN) and 3,178 cases of venous invasion (including 251 cases of PN and 2,927 cases of RN). The demographics and clinicopathological data of the RN group and the PN group in the two cohorts showed significant differences (**Tables 2**, **3**). KMA showed that the overall survival (OS) and CSS of the PN groups were favorable than those of the RN groups in the two cohorts (both *p* < 0.001) (**Figure 2**). After PSM, there was no significant difference in the characteristics between the PN and RN groups in the two cohorts (**Tables 2**, **3**). There was no significant difference in OS (*p* = 0.068) and CSS (*p* = 0.190) between the PN and RN groups in the venous invasion cohort. The OS in the fat invasion group was favorable in the PN group (*p* = 0.036), while CSS was comparable in the two groups (*p* = 0.075) (**Figure 3**).

**Table 2:** Characteristics of fat invasion cohort before and after PSM.

Characteristics	Before match			After match		
	PN (n = 986)	RN (n = 2,963)	*p*	PN (n = 496)	RN (n = 496)	*p*
Year of diagnosis (median [IQR])	2,013.00 [2,012.00, 2,014.00]	2,013.00 [2,011.00, 2,014.00]	<0.001	2,013.00 [2,012.00, 2,014.00]	2,013.00 [2,011.00, 2,014.00]	0.977
Age (median [IQR])	62.00 [57.00, 72.00]	62.00 [57.00, 72.00]	0.140	62.00 [57.00, 72.00]	67.00 [57.00, 72.00]	0.274
Size (median [IQR])	38.00 [27.00, 50.00]	70.00 [50.00, 92.00]	<0.001	38.00 [26.00, 50.00]	40.00 [28.00, 50.00]	0.503
Grade (%)			<0.001			0.892
I	59 (6.0)	105 (3.5)		26 (5.2)	29 (5.8)	
II	419 (42.5)	1,010 (34.1)		212 (42.7)	200 (40.3)	
III	306 (31.0)	1,097 (37.0)		144 (29.0)	156 (31.5)	
IV	61 (6.2)	343 (11.6)		38 (7.7)	35 (7.1)	
Unknown	141 (14.3)	408 (13.8)		76 (15.3)	76 (15.3)	
Race (%)			0.042			0.437
Black	93 (9.4)	220 (7.4)		50 (10.1)	50 (10.1)	
Other	75 (7.6)	189 (6.4)		38 (7.7)	28 (5.6)	
White	818 (83.0)	2,554 (86.2)		408 (82.3)	418 (84.3)	
Sex (%)			0.275			0.186
Female	274 (27.8)	879 (29.7)		136 (27.4)	156 (31.5)	
Male	712 (72.2)	2,084 (70.3)		360 (72.6)	340 (68.5)	
Histology (%)			<0.001			0.975
CCRCC	563 (57.1)	2,104 (71.0)		282 (56.9)	288 (58.1)	
CHRCC	108 (11.0)	241 (8.1)		57 (11.5)	53 (10.7)	
PRCC	227 (23.0)	253 (8.5)		107 (21.6)	105 (21.2)	
Others	88 (8.9)	365 (12.3)		50 (10.1)	50 (10.1)	
Laterality (%)			0.040			0.949
Left	470 (47.7)	1,525 (51.5)		245 (49.4)	247 (49.8)	
Right	516 (52.3)	1,438 (48.5)		251 (50.6)	249 (50.2)	
Chemotherapy (%)			<0.001			0.328
No/unknown	974 (98.8)	2,850 (96.2)		490 (98.8)	485 (97.8)	
Yes	12 (1.2)	113 (3.8)		6 (1.2)	11 (2.2)	

PSM, propensity score matching; PN, partial nephrectomy; RN, radical nephrectomy; IQR, interquartile range; CCRCC, clear cell renal cell carcinoma; CHRCC, chromophobe renal cell carcinoma; PRCC, papillary renal cell carcinoma.

**Table 3:** Characteristics of venous invasion cohort before and after PSM.

Characteristics	Before match			After match		
	PN (n = 251)	RN (n = 2,927)	*p*	PN (n = 251)	RN (n = 251)	*p*
Year of Diagnosis (median [IQR])	2,013.00 [2,011.00, 2,014.00]	2,013.00 [2,011.00, 2,014.00]	0.566	2,013.00 [2,011.00, 2,014.00]	2,013.00 [2,011.00, 2,014.00]	0.937
Age (median [IQR])	62.00 [57.00, 72.00]	62.00 [57.00, 72.00]	0.058	62.00 [57.00, 72.00]	62.00 [57.00, 72.00]	0.515
Size (median [IQR])	46.00 [35.00, 67.50]	76.00 [57.00, 100.00]	<0.001	46.00 [35.00, 67.50]	50.00 [35.00, 70.00]	0.741
Grade (%)			<0.001			0.890
I	9 (3.6)	61 (2.1)		9 (3.6)	12 (4.8)	
II	89 (35.5)	936 (32.0)		89 (35.5)	83 (33.1)	
III	108 (43.0)	1,206 (41.2)		108 (43.0)	111 (44.2)	
IV	12 (4.8)	420 (14.3)		12 (4.8)	15 (6.0)	
Unknown	33 (13.1)	304 (10.4)		33 (13.1)	30 (12.0)	
Race (%)			0.602			1.000
Black	18 (7.2)	177 (6.0)		18 (7.2)	18 (7.2)	
Other	13 (5.2)	188 (6.4)		13 (5.2)	14 (5.6)	
White	220 (87.6)	2,562 (87.5)		220 (87.6)	219 (87.3)	
Sex (%)			1.000			0.774
Female	78 (31.1)	917 (31.3)		78 (31.1)	82 (32.7)	
Male	173 (68.9)	2,010 (68.7)		173 (68.9)	169 (67.3)	
Histology (%)			0.057			0.839
CCRCC	203 (80.9)	2,440 (83.4)		203 (80.9)	200 (79.7)	
CHRCC	11 (4.4)	71 (2.4)		11 (4.4)	9 (3.6)	
PRCC	11 (4.4)	70 (2.4)		11 (4.4)	10 (4.0)	
Others	26 (10.4)	346 (11.8)		26 (10.4)	32 (12.7)	
Laterality (%)			0.948			0.858
Left	128 (51.0)	1,485 (50.7)		128 (51.0)	131 (52.2)	
Right	123 (49.0)	1,442 (49.3)		123 (49.0)	120 (47.8)	
Chemotherapy (%)			0.315			0.531
No/unknown	241 (96.0)	2,759 (94.3)		241 (96.0)	237 (94.4)	
Yes	10 (4.0)	168 (5.7)		10 (4.0)	14 (5.6)	

PSM, propensity score matching; PN, partial nephrectomy; RN, radical nephrectomy; IQR, interquartile range; CCRCC, clear cell renal cell carcinoma; CHRCC, chromophobe renal cell carcinoma; PRCC, papillary renal cell carcinoma.

Univariate and multivariate regression analyses showed that the risk factors of OS in the fat invasion cohort were higher age (hazard ratio (HR) 1.05, *p* < 0.001), greater tumor diameter (HR 1.01, *p* = 0.007), surgical method was RN (HR 1.35, *p* = 0.040), and earlier year of diagnosis (HR 1.10, *p* = 0.032); nuclear grade is also an independent prognostic factor (*p* = 0.016) (**Table 4**). The risk factors for CSS in the fat invasion cohort were higher age (HR 1.05, *p* < 0.001), greater tumor diameter (HR 1.02, *p* = 0.007), and underwent systemic therapy (HR 3.75, *p* = 0.002); and pathological grade was also an independent prognostic factor (*p* < 0.001) (**Table 5**). Higher age was risk factor of both OS (HR 1.05, *p* < 0.001) and CSS (HR 1.04, *p* = 0.001) in the venous invasion cohort (**Tables 6**, **7**). The surgical method had no significant effect on the OS (*p* = 0.069) and CSS (*p* = 0.190) of the venous invasion cohort and had no significant effect on the CSS (*p* = 0.078) of the fat invasion cohort (**Table 8**).

**Table 4:** Univariate and multivariate analyses of OS in the fat invasion cohort.

Characteristics	Univariable analysis			Multivariable analysis		
	Hazard ratio	CI95.	*p*-Value	Hazard ratio	CI95.	*p*-Value
Chemotherapy						
No/unknown	1 (Reference)			1 (Reference)		
Yes	2.33	1.09–4.95	0.028	2.09	0.97–4.50	0.06
Grade			0.001			0.016
I	1 (Reference)			1 (Reference)		
II	1.78	0.77–4.09	0.175	1.59	0.69–3.67	0.275
III	2.4	1.04–5.53	0.04	1.83	0.79–4.25	0.161
IV	4.22	1.72–10.33	0.002	3.49	1.41–8.63	0.007
Unknown	1.74	0.72–4.24	0.22	1.58	0.65–3.85	0.315
Size	1.01	1.00–1.02	<0.001	1.01	1.00–1.01	0.007
Surgery						
PN	1 (Reference)			1 (Reference)		
RN	1.35	1.02–1.79	0.037	1.35	1.01–1.79	0.04
Year of diagnosis (per year)	0.91	0.84–1.00	0.049	0.91	0.83–0.99	0.032

OS, overall survival; PN, partial nephrectomy; RN, radical nephrectomy.

**Table 5:** Univariate and multivariate analyses of CSS in the fat invasion cohort.

Characteristics	Univariable analysis			Multivariable analysis		
	Hazard ratio	CI95.	*p*-Value	Hazard ratio	CI95.	*p*-Value
Chemotherapy						
No/unknown	1 (Reference)			1 (Reference)		
Yes	5.21	2.26–11.98	<0.001	3.75	1.6–8.78	0.002
Grade			<0.001			<0.001
I	1 (Reference)			1 (Reference)		
II	1.42	0.33–6.08	0.635	1.14	0.27–4.91	0.856
III	3.47	0.83–14.45	0.088	2.1	0.5–8.87	0.315
IV	8.6	1.98–37.27	0.004	5.43	1.24–23.72	0.025
Unknown	1.18	0.24–5.86	0.837	0.94	0.19–4.66	0.938
Size (per mm)	1.02	1.01–1.03	<0.001	1.02	1.01–1.02	<0.001

CSS, cause-specific survival.

**Table 6:** Univariate and multivariate analyses of OS in the venous invasion cohort.

Characteristics	Univariable analysis			Multivariable analysis		
	Hazard ratio	CI95.	*p*-Value	Hazard ratio	CI95.	*p*-Value
Age (per year)	1.05	1.03–1.07	<0.001	╱	╱	╱

OS, overall survival.

**Table 7:** Univariate and multivariate analyses of CSS in the venous invasion cohort.

Characteristics	Univariable analysis			Multivariable analysis		
	Hazard ratio	CI95.	*p*-Value	Hazard ratio	CI95.	*p*-Value
Grade			0.013			0.055
I	1 (Reference)			1 (Reference)		
II	0.41	0.13–1.27	0.124	0.48	0.15–1.48	0.199
III	0.85	0.3–2.43	0.762	0.81	0.28–2.34	0.699
IV	1.73	0.52–5.76	0.37	1.58	0.47–5.27	0.46
Unknown	0.38	0.1–1.54	0.177	0.36	0.09–1.48	0.158
Size (per mm)	1.01	1–1.02	0.018	1.01	1–1.02	0.089

CSS, cause-specific survival.

**Table 8:** Association of nephrectomy type and survival (partial nephrectomy is reference).

Subgroup	CSS						OS					
	Univar			Multivar			Univar			Multivar		
	HR	95%CI	*p*	HR	95%CI	*p*	HR	95%CI	*p*	HR	95%CI	*p*
Fat invasion cohort	1.5	0.96–2.36	0.078	╱	╱	╱	1.35	1.02–1.79	0.037	1.35	1.01–1.79	0.04
VTT invasion cohort	1.4	0.84–2.33	0.19	╱	╱	╱	1.43	0.97–2.10	0.069	╱	╱	╱

CSS, cause-specific survival; OS, overall survival; HR, hazard ratio; VTT, venous tumor thrombus.

**Figure 4:**
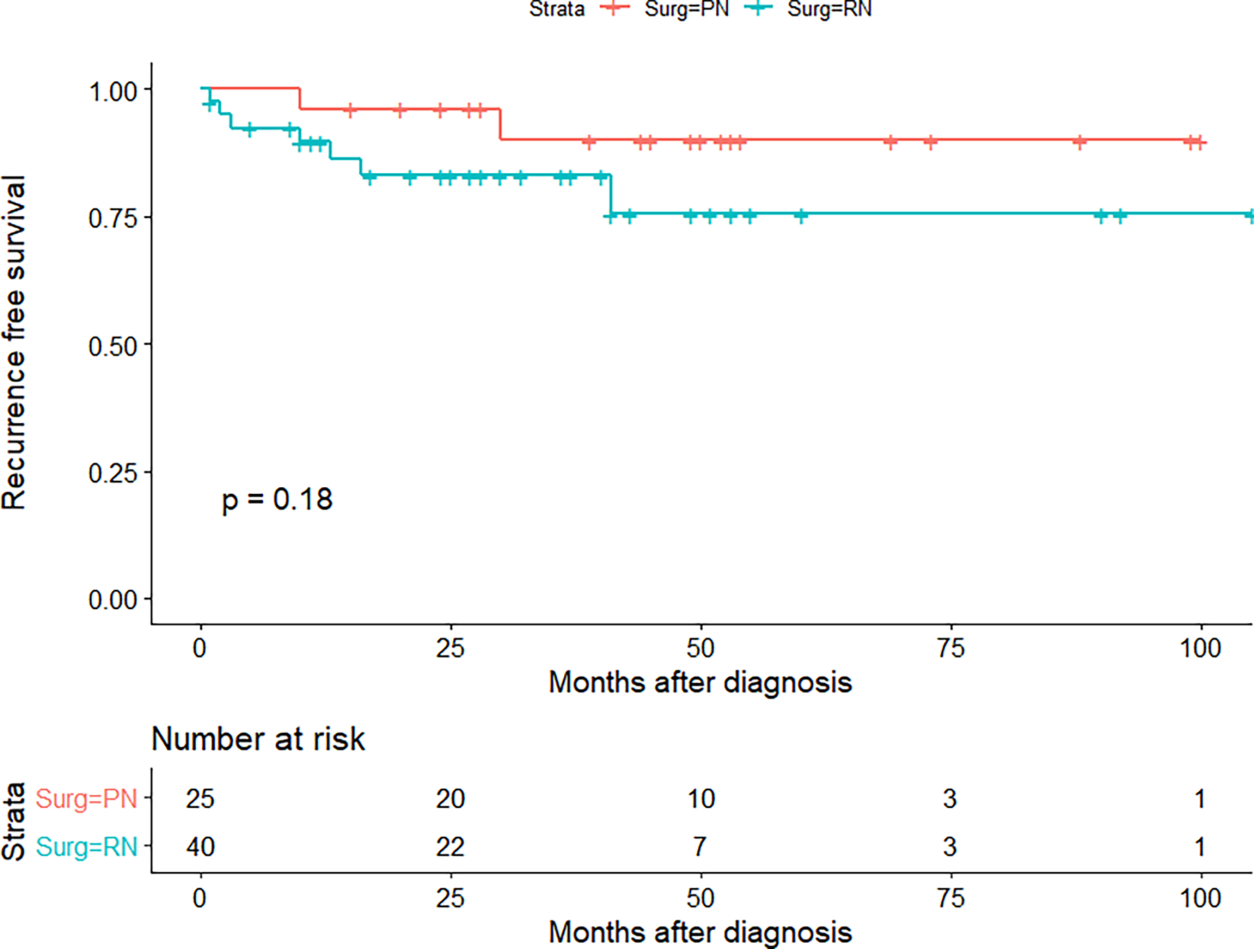
Recurrence-free survival (RFS) curve of partial nephrectomy (PN) and radical nephrectomy (RN) group in Tongji Hospital (TJH) cohort.

The median age of the TJH cohort was 53 (26–73) years; the TJH cohort included 57 (87.7%) fat invasion and 8 (12.3%) venous invasion patients, and 25 (38.5%) PN and 40 (61.5%) RN patients (**Table 9**). Postoperative estimated glomerular filtration rate (eGFR) was greater in patients receiving PN (*p* = 0.027). The median follow-up was 30 (1–105) months, 5 patients died at the last follow-up (all of them died of RCC), and 9 cases underwent cancer relapsed. The 1-, 3-, and 5-year OS rates (CSS same as OS) of the TJH cohort were 93.6%, 91.8%, and 91.8%, respectively; the 1-, 3-, and 5-year RFS rates were 90.2%, 85.7%, and 82.2%, respectively. KMA showed no significant difference in RFS between the RN and PN groups (*p* = 0.170) (**Figure 4**).

**Table 9:** Characteristics of PN and RN groups in TJH cohorts.

Characteristics	PN (*p* = 25)	RN (*p* = 40)	*p*
Sex			0.290
Female	6 (24.0)	15 (37.5)	
Male	19 (76.0)	25 (62.5)	
Age (median [IQR])	53.00 [46.00, 59.00]	52.50 [45.75, 62.00]	0.766
BMI (median [IQR])	25.00 [23.02, 26.49]	23.29 [21.60, 25.72]	0.124
ASA score			0.698
High	2 (8.0)	5 (12.5)	
Low	23 (92.0)	35 (87.5)	
Hemoglobin (median [IQR])	144.00 [138.00, 150.00]	124.50 [113.75, 143.25]	0.001
Albumin (median [IQR])	41.90 [39.30, 43.70]	40.15 [38.15, 42.18]	0.060
Serum_Cr (median [IQR])	80.00 [66.00, 93.00]	80.50 [66.50, 96.00]	0.777
Preoperative eGFR (median [IQR])	89.00 [79.40, 100.60]	90.45 [76.00, 103.60]	0.824
Postoperative eGFR (median [IQR])	78.90 [57.30, 86.70]	60.2 [51.10, 68.35]	0.027
Grade			0.120
High	4 (16.0)	15 (37.5)	
Low	17 (68.0)	17 (42.5)	
Unknown	4 (16.0)	8 (20.0)	
RENAL_score			0.015
High	3 (12.0)	6 (15.0)	
Low	9 (36.0)	3 (7.5)	
Moderate	13 (52.0)	31 (77.5)	
Laterality			0.799
Left	11 (44.0)	20 (50.0)	
Right	14 (56.0)	20 (50.0)	
Approach			<0.001
Laparoscopic	10 (40.0)	27 (67.5)	
Open	0 (0.0)	7 (17.5)	
Robotic	15 (60.0)	6 (15.0)	
Histology			0.579
CCRCC	19 (76.0)	27 (67.5)	
Non-CCRCC	6 (24.0)	13 (32.5)	
Subgroup			0.139
Fat invasion	24 (96.0)	33 (82.5)	
Venous invasion	1 (4.0)	7 (17.5)	
Renal failure			1.000
No	24 (96.0)	38 (95.0)	
Yes	1 (4.0)	2 (5.0)	

PN, partial nephrectomy; RN, radical nephrectomy; TJH, Tongji Hospital; IQR, interquartile range; BMI, body mass index; ASA, American Society of Anesthesiologists; eGFR, estimated glomerular filtration rate; CCRCC, clear cell renal cell carcinoma.

## Discussion

With the progression of the technology and the in-depth understanding of RCC, the indications for PN are gradually expanding, from initial T1a to T1b and then to the current selected T2 (5, 16). It is reasonable to believe that PN will be provided to some appropriate T3a patients in some technologically advanced institutions (12).

Even though urologists do not deliberately provide PN for T3a patients, they will encounter cases where cT1 and cT2 patients undergo PN upstage to pT3a. The postoperative upstaging occurs in approximately 11.35% of cT1 and cT2 patients; studies have shown that RN does not improve the RFS of upstaged patients as compared with PN (9). The study of Russell et al. included 1,955 cases with cT1 RCC who underwent PN, 95 of which upstaged to pT3a, and the PFS and CSS of pT3a patients were significantly worse than those of pT1 patients (both *p* < 0.01) (17). The study of Groin et al. included 855 RCC patients who received PN, 41 (4.8%) of them upstaged to pT3a, and the recurrence rate of pT3a patients was significantly higher than that of pT1–2 patients at 2 years (99.2% vs. 91.8%) (18); these studies supported the current T staging but did not compare the prognosis of T3a patients with PN or RN. Shvero et al. compared 48 pT3a RCC patients who received PN with 86 patients who received RN and found that the surgical method was not significantly related to local recurrence, distant metastasis, CSS, or OS (10). Research by Andrade et al. and Deng et al. also drew similar conclusions (10, 19), but the study of Shah et al. showed that pT3a patients receiving PN are associated with shorter RFS (*p* = 0.001) (14). In our study’s SEER cohort and the TJH cohort, the CSS or RFS of pT3a RCC patients who received PN was not worse than that of patients who received RN.

In addition to upstaged pT3a patients, some institutions have also tried to actively perform PN for cT3a patients. The study by Yim et al. included 159 cT3a RCC patients who received robot-assisted PN from multiple centers, of which 64.3% of the cases achieved a trifecta (negative surgical margins, warm ischemia time (WIT) ≤ 25 min, and no perioperative complications), and 37.6% of patients achieved the optimal outcome (trifecta and ≥90% preservation of the eGFR and no stage upgrading of chronic kidney disease). The 5-year RFS, CSS, and OS were 82.1%, 93.3%, and 91.3%, respectively. The downside is that there is no RN patient as a control (12).

Compared with RN, PN is positively correlated with the risk of having a positive surgical margin, and about 2%–8% of PN patients have a positive surgical margin (20). Morris et al. found that T3 RCC patients with positive margins after RN showed a trend of poorer OS, but it was not statistically significant (21). The study by Petros et al. showed that positive margins were associated with recurrence, metastasis, and worse OS in PN patients (22). However, Tabayoyong et al., Takagi et al., and Kang et al. found that positive margins in patients with PN are not certainly translated into worse oncologic outcomes (23–25).

Some studies found that patients who received PN had better RFS and CSS than those who received RN and interpreted it as greater renal function preservation, which might be related to better oncologic outcomes (26, 27); the same trend was also observed in the TJH cohort in our study. Palacios et al. found that the unfavorable oncologic outcome was more related to the aggressive characteristics of the tumor itself, rather than the degree of renal function preservation (28). In our study, PN in the pre-PSM cohort was also significantly correlated with better CSS, but it was no longer significant after PSM. Therefore, we should cautiously interpret the trend that CSS and RFS of PN patients are better than those of RN patients in the study, which may be caused by retrospective study design and selection bias.

In view of the fact that some information such as hemoglobin, albumin, BMI, and comorbidities cannot be obtained from the SEER database, there were also some biases in this study, which may affect the accuracy of the conclusion.

## Conclusions

This study proved that PN is safe and feasible in localized T3a RCC patients *via* a retrospective study with a large sample volume, and the oncologic outcomes of patients who underwent PN were comparable with those of patients who received RN but limited to pT3a patients, and higher-quality research is needed before exploring performing PN for cT3a RCC patients.

## Data Availability Statement

The raw data supporting the conclusions of this article will be made available by the authors, without undue reservation.

## Ethics Statement

The studies involving human participants were reviewed and approved by the Medical Ethical Committee of Tongji Hospital of Huazhong University of Science and Technology. Written informed consent for participation was not required for this study in accordance with the national legislation and the institutional requirements.

## Author Contributions

JT: data curation, writing—conceptualization, and original draft. XZ: formal analysis and supervision. JW: data curation and data analysis. JG: data curation and writing—review and editing. CK: software and supervision. WG: data analysis and supervision. ZH: project administration, resources, supervision, and funding acquisition. CY: writing—conceptualization, funding acquisition, and writing—review and editing. All authors contributed to the article and approved the submitted version.

## Conflict of Interest

The authors declare that the research was conducted in the absence of any commercial or financial relationships that could be construed as a potential conflict of interest.

## Publisher’s Note

All claims expressed in this article are solely those of the authors and do not necessarily represent those of their affiliated organizations, or those of the publisher, the editors and the reviewers. Any product that may be evaluated in this article, or claim that may be made by its manufacturer, is not guaranteed or endorsed by the publisher.
